# Exploring Metal Interactions with Released Polysaccharides from *Cyanothece* sp. CE4: A Chemical and Spectroscopic Study on Biosorption Mechanism

**DOI:** 10.3390/polym17030371

**Published:** 2025-01-29

**Authors:** Matilde Ciani, Giovanni Orazio Lepore, Alessandro Puri, Giorgio Facchetti, Alessandra Adessi

**Affiliations:** 1 Department of Agriculture, Food, Environment and Forestry (DAGRI), University of Florence, 50144 Florence, Italy; matilde.ciani@unifi.it; 2 Department of Earth Science, University of Florence, 50121 Florence, Italy; giovanniorazio.lepore@unifi.it; 3 Department of Physics and Astronomy, Alma Mater Studiorum, University of Bologna, 40127 Bologna, Italy; alessandro.puri@unibo.it; 4 CNR-IOM-OGG c/o ESRF, The European Synchrotron, 38043 Grenoble, France; 5 Department of Pharmaceutical Sciences (DISFARM), University of Milan, 20133 Milan, Italy; giorgio.facchetti@unimi.it

**Keywords:** circular resource management, cyanobacteria, exopolysaccharides, metal biosorption, X-ray absorption spectroscopy

## Abstract

This study investigates the potential of released polysaccharides (RPS) from the halophilic cyanobacterium *Cyanothece* sp. CE4 as biosorbents for heavy metals, specifically copper (Cu), nickel (Ni), and zinc (Zn). By combining ICP-OES, SEM-EDX, FT-IR spectroscopy, and XAS techniques, this work provides a comprehensive chemical and spectroscopic analysis of the biosorption mechanisms driving metal removal. The results revealed a strong binding affinity for Cu, followed by Ni and Zn, with RPS functional groups playing a key role in metal coordination. The RPS efficiently removed metals from both monometallic and multimetallic solutions, emphasizing their adaptability in competitive environments. XAS analysis highlighted unique metal-specific coordination patterns. Ni preferentially binds to oxygen donors and Zn to chlorine, and Cu exhibits non-selective binding. Remarkably, the extracted RPS achieved a maximum Cu removal capacity of 67 mg per gram of RPS dry weight, surpassing previously reported biosorption capacities. This study not only advances the understanding of biosorption mechanisms by cyanobacterial RPS but also emphasizes their dual role in environmental remediation and circular resource management. The insights provided here establish a foundation for the development of sustainable, cyanobacteria-based solutions for heavy-metal recovery and environmental sustainability.

## 1. Introduction

Cyanobacteria, a group of photoautotrophic bacteria, have gained significant attention for their potential in environmental applications, particularly in the field of bioremediation [1,2]. Among their many capabilities, the production of exopolysaccharides (EPS) stands out as a key feature in facilitating the sequestration and removal of heavy metals from polluted environments [1]. Cyanobacteria produce various forms of EPS, which can be cell-bound, including capsular EPS, sheaths and slimes, or released EPS (released polysaccharides, RPS) [3]. The differences between these forms are significant in terms of structure and function. Capsular EPS are tightly associated with the cell wall, providing protection and playing a minor role in metal uptake, while sheaths are thicker layers that enclose the cells, offering additional defense against external stressors [4]. Slime EPS, often loosely attached, are more involved in maintaining moisture and nutrient balance in cyanobacterial biofilms [4]. However, RPSs exhibit high efficiency in metal biosorption due to their mobility and high exposure to metal ions in solution [5,6]. Indeed, cyanobacterial EPSs are chemically complex, comprising polysaccharides with functional groups such as carboxyl, hydroxyl, sulfate, and phosphate. These groups allow EPSs to interact with other ions, including metals, forming stable complexes that facilitate the removal of contaminants from aqueous environments [7,8]. Their ability to bind not only metals but also organic pollutants makes them key agents in the development of bioremediation strategies for diverse contaminants. In the context of metal remediation, the biosorption affinity and efficiency are influenced by the chemical composition of the EPS, which varies between species and environmental conditions. For instance, EPS rich in negatively charged groups, such as carboxyl and sulfate, show a higher affinity for positively charged metal ions like copper (Cu), nickel (Ni), and zinc (Zn), forming stable metal complexes [9,10,11]. The concentration of EPS also affects biosorption efficiency. Higher EPS production typically correlates with greater metal removal efficiency, as more binding sites become available for interaction with metal ions [10,12].

Beyond their role in biosorption, cyanobacteria hold considerable promise within the framework of a circular economy, where waste materials are transformed into valuable products [13]. A key advantage of using the RPS-containing supernatant from a cyanobacterial culture for metal biosorption, as opposed to the whole biomass, lies in the possibility of valorizing the residual cellular biomass for other applications. By isolating the supernatant for metal removal, the remaining biomass can be repurposed in a biorefinery context, offering opportunities for pigment extraction (e.g., phycobiliproteins), protein production, and bioenergy generation through processes such as anaerobic digestion or bioethanol production [6,14]. Also, it reduces the need for biomass regeneration, since the cells obtained after fraction separation can serve as feedstock for continuous RPS generation [13,15,16].

Considering the critical role of EPS in biosorption, this study investigates the interaction properties between metals and RPS produced by the halophilic cyanobacterium *Cyanothece* sp. CE4. The RPS-containing supernatant was employed to assess its capacity for removing Cu, Ni, and Zn from aqueous solutions. Using a suite of chemical and spectroscopic techniques, this study examines the interactions between RPS and metals, elucidating the mechanisms of binding and coordination. To confirm the pivotal role of RPS in metal uptake, isolated and extracted RPS from the supernatant were evaluated at varying concentrations as Cu biosorbents. This research aims to provide deeper insights into the strain-specific metal interaction capabilities of *Cyanothece* sp. CE4, highlighting its dual functionality as a biosorbent and a contributor to sustainable resource management.

## 2. Material and Methods

### 2.1. Experimental Plan

The cyanobacterium *Cyanothece* sp. CE4 (Genbank: OQ945752) was selected for its ability to remove metals and produce EPS, as previously described in Ciani et al. [12]. The cyanobacterium was cultivated for one week in 1-L bubbled flat glass bottles containing a seawater medium enriched as follows (g L^−1^): NaNO_3_, 1.5; Na_2_HPO_4_, 0.04; NaHCO_3_, 0.1; NaCl, 28; ferric ammonium citrate, 0.006; citric acid, 0.006; Na_2_ EDTA, 0.001; and 0.5 mL L^−1^ of trace metal solution. The bottles were bubbled with a sterile air/CO_2_ mixture (99:1, v:v) at a flow rate of 0.2 L per L of culture per minute with continuous light provided by an LED lamp with 200 µmol photons m^−2^ s^−1^. Then, the biomass was centrifuged at 4000 rpm for 15 min, and the supernatant containing the RPS was collected. First, an experiment adopting the RPS-containing supernatant experiment was carried out to investigate the interaction with bivalent metals (i.e., Cu, Ni, and Zn), as described in Section 2.2. The metal content in the solutions was 10 mg L^−1^ for each metal, reaching a total amount of 30 mg L^−1^ in the multimetallic solutions, obtained from stock solutions of 1 g L^−1^ Cu, Ni, and Zn using their respective chloride salts (CuCl_2_, NiCl_2_, and ZnCl_2_). The RPS-containing supernatant obtained after the exposure to mono- and multimetallic solutions was collected and freeze-dried to investigate its interaction with the metals adopting ICP-OES (inductively coupled plasma optical emission spectroscopy), SEM-EDX (scanning electron microscopy with energy dispersive X-ray analysis), FT-IR (Fourier-transform infrared) spectroscopy, XANES (X-ray absorption near-edge structure), and EXAFS (extended X-ray absorption fine structure) spectroscopy. Then, to confirm the role of RPS in metal biosorption, a further experiment, named the extracted-RPS experiment, was carried out as detailed in Section 2.3. Thus, extracted RPS at different concentrations were adopted as Cu biosorbents.

### 2.2. RPS-Containing Supernatant Experiment

The supernatant collected from a one-week grown culture was confined in dialysis tubings (Medicell Membranes Ltd., London, UK, 12–14,000 MWCO) and pre-treated with an acidic solution (HCl 0.1M), as previously described in Ciani et al. [12]. The same pre-treatment was carried out for the dialysis tubings containing distilled water and used as a blank.

Pre-treated samples were dipped into Cu, Ni, and Zn mono- and multimetallic solutions for treated samples and the blank or distilled water for the control and kept under continuous mixing, as previously described [12]. The contact with monometallic and multimetallic solutions was carried out in duplicate. After 24 h of contact, the metallic solutions were removed, and the confined samples enriched in metals were collected and freeze-dried for further characterization.

#### Analytical Procedures

Cu, Ni, and Zn concentrations were detected in freeze-dried samples, previously digested with HNO_3_, using ICP-OES (iCAP 7400 ICP-OES Analyzer, Thermo Fisher Scientific, Waltham, MA, USA following the APAT CNR IRSA 3020 Man 29 2003 protocol. Fourier-transform infrared (FTIR) spectra were acquired in all the samples, including the control, to identify the functional groups of the RPS-containing supernatant possibly involved in the binding with metal. FTIR spectroscopy was conducted with a Perkin Elmer FTIR Spectrometer “Spectrum One” (Perkin Elmer, Waltham, MA, USA) across a spectral range of 4000–650 cm^−1^, using the transmittance mode with 32 scans per sample and a resolution of 4 cm^−1^.

Additionally, the structural and elemental features were examined via SEM-EDX on freeze-dried samples mounted on carbon tape and coated with a gold–palladium layer to enhance conductivity. The SEM analysis of the samples obtained after exposure to multimetallic solutions was performed using a SIGMA 500/VP microscope (Carl ZEISS, Oberkochen, Germany). SEM images were captured at 3.00K magnification with an accelerating voltage of 20 kV, and a scale of 10 µm was used for elemental composition analysis with EDX (ZEISS EVO 15, Carl ZEISS, Oberkochen, Germany). SEM-EDX observations of the samples exposed to monometallic solutions were carried out at the European Synchrotron Radiation Facility (ESRF, Grenoble, France) adopting a Zeiss LEO 1530 high-resolution scanning electron microscope coupled with a Schottky field-emission gun that is equipped with secondary electron, in-lens, and backscattered detectors for imaging. The analysis was carried out with a resolution of 1 nm at 20 keV voltage. An analysis system from Oxford Instruments was used for element analysis. The INCA V7 microanalysis software is coupled with a 20 mm^2^ large-area silicon drift detector (SDD) for fast elemental mapping.

XAS investigations of Cu, Ni, and Zn at their K-edges were carried out at the BM08-LISA CRG beamline [17] at the European Synchrotron Radiation Facility (ESRF, Grenoble, France). Freeze-dried samples were mounted on carbon tabs, covered with Kapton foil to prevent sample loss, and placed on aluminum sample holders. Cellulose pellets were prepared for the model compounds (e.g., CuCl_2_, Cu(CO_2_CH_3_)_2_, CuO, Cu_2_O, CuSO_4_, ZnCl_2_, Zn(CO_2_CH_3_)_2_, ZnO, ZnSO_4_, NiCl_2_, Ni(CO_2_CH_3_)_2_, and NiO). The measurements employed a non-focused beam (~1 mm × 2 mm) with Si(311) monochromator crystals, and Si mirrors were used for harmonics rejection (E_cutoff ≈ 15 KeV). The estimated photon flux on the sample was ~8 × 10^9^ photons/s. XAS spectra were collected in fluorescence mode using a Si photodiode for the samples or in transmission mode for the model compounds, with the measurements conducted under vacuum at 80 K to minimize beam damage. The XANES region was scanned with a step size of 0.5 eV, while the pre-edge region was sampled at 0.15 eV intervals. The post-edge EXAFS region was collected with a fixed k step width of 0.05 Å^−1^ up to a maximum k value of 16 Å^−1^. Data analysis was performed using ATHENA software (version 0.8.061) [18] for background subtraction, edge step normalization, energy calibration, and averaging of spectra. Normalized XANES spectra of samples and reference compounds were compared to estimate metal oxidation states, and structural EXAFS signals (k·χ (k)) were extracted. Fits were conducted using ARTEMIS software (version 0.8.014) [18], with the amplitude reduction factor (S_0_^2^) fixed to the value obtained from model compound fits, except for Ni samples. Model atomic clusters centered on the absorber atom were generated using the online version of ATOMS [19], and theoretical amplitude and phase functions were calculated with the FEFF8 code [19,20]. To differentiate scatterers with similar atomic numbers, such as N/O and S/Cl, coordination numbers were determined using the bond valence model [21], following the procedure outlined by Marmiroli et al. [22]. This approach considers the relationship between bond length, bond valence, and coordination, incorporating a ligand-dependent parameter obtained from the literature [23,24,25].

### 2.3. Extracted RPS Experiment

To identify the role of RPS in the soluble fraction, a further test was carried out by adopting RPS extracted from the supernatant of a one-week-grown *Cyanothece* sp. CE4 culture. RPS were isolated by centrifuging the culture at 4000 rpm for 30 min at room temperature. The supernatant was then transferred into nitrocellulose tubular dialysis membranes (Medicell International, 12–14,000 MWCO) and dialyzed against 10 volumes of distilled water for 48 h under continuous stirring, with two water changes during this period. Following dialysis, the RPSs were concentrated using an orbital evaporator at 35 °C. The RPSs were subsequently precipitated by mixing with two volumes of isopropyl alcohol and incubated at 4 °C overnight. After centrifugation at 4000 rpm for 15 min, the resulting PS pellets were resuspended in distilled water according to Moia et al. [26] and then freeze-dried. The sulfate content of the RPS was determined by an adaptation of Dogson and Yu et al. [27,28]. Briefly, 2 mg of RPS were hydrolyzed with 1 mL of HCl 4 M for 2 h at 120 °C. The samples were centrifuged, and 100 µL of supernatant were added to 1.9 mL of TCA 3% and 0.5 mL of Barium chloride–gelatin reagent and vortexed. After 15 min, the samples were measured with a UV-Vis spectrophotometer at 360 nm.

Freeze-dried RPSs were dissolved in distilled water at 0.25, 0.50, 0.75, and 1.00 g L^−1^ to evaluate the effect of RPS concentration in Cu removal. Cu was selected as the standard metal due to the higher removal efficiency exhibited during the previous study. The samples were confined into dialysis tubings and pre-treated as described in Section 2.2. before being exposed to the 10 mg L^−1^ Cu solution. After 24 h of contact, the remaining Cu content in the solution was quantified by adopting an adaptation of the bicinchoninate method (HI 93,702 kit, Hanna Instruments Srl, Padova, Italy) following the manufacturer’s instructions. Cu removal efficiency (% of Cu removed) and Cu specific uptake (mg Cu removed per gram of RPS dry weight) were calculated by comparing the Cu content of the solutions treated with the RPS and the solutions treated with distilled water confined in dialysis tubings and pre-treated like the RPS. The effect of RPS concentration on Cu biosorption was analyzed with a one-way ANOVA and Tukey’s test at the 5 % significance level by adopting Origin Pro software (version 2023.b).

## 3. Results and Discussion

### 3.1. RPS-Containing Supernatant Experiment

Multiple analytical techniques, including SEM-EDX, FT-IR, and XAS spectroscopy, were adopted to investigate the interaction between Cu, Ni, and Zn and the RPS from *Cyanothece* sp. CE4 during the metal biosorption process.

**Figure 1:**
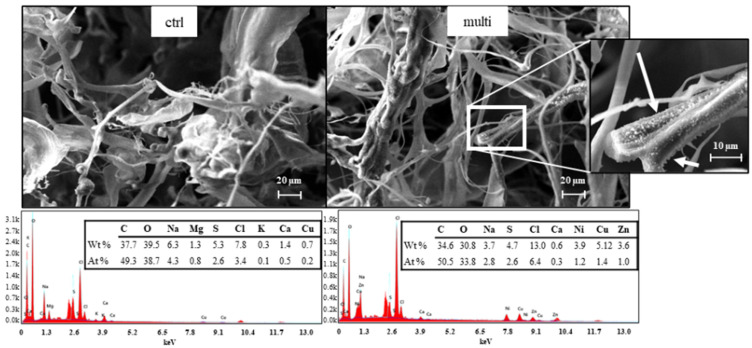
SEM micrographs of RPS-containing supernatant after contact with distilled water (control, on the left, 1000× magnification) and multimetallic solution (multi-sample, on the right, 1000 and 3000× magnitude) coupled with EDX spectra and results expressed as atomic and weight percentage shown below each micrograph. White arrows indicate white agglomerations possibly due to metal deposits.

**Figure 2:**
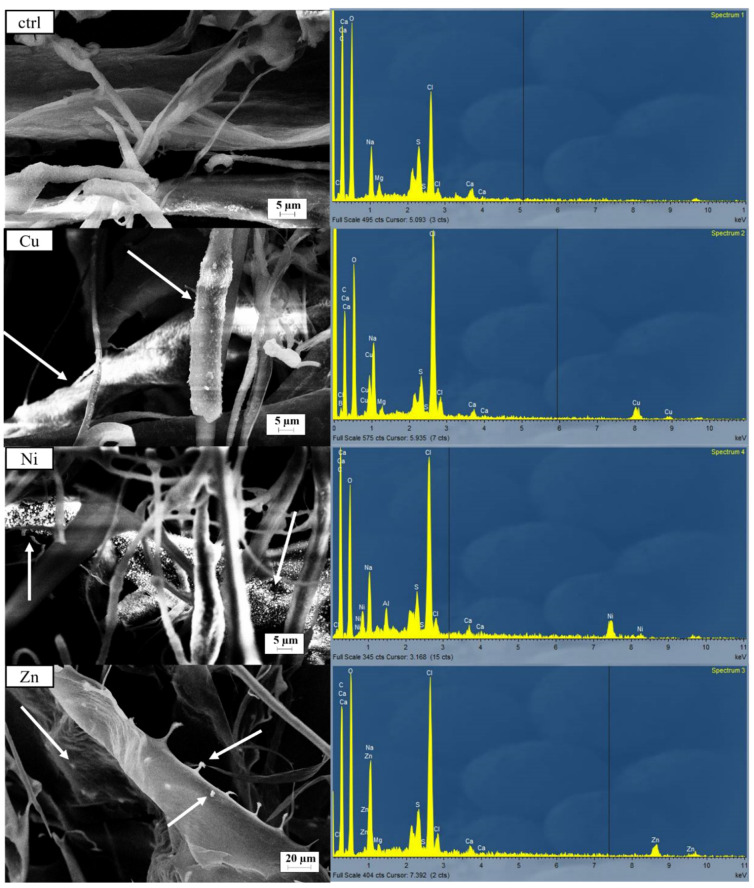
SEM micrographs of RPS-containing supernatant after contact (in order from the top) with distilled water (ctrl), Cu, Ni, and Zn solutions at 1700×, 1500×, 700×, and 400× magnification, respectively, coupled with EDX microanalysis shown on the right of each micrograph. White arrows indicate white agglomerations possibly due to metal accumulation.

The metal content in the RPS-containing supernatant was quantified after exposure to metallic solutions or distilled water (control). The table below (Table 1) shows the metal content in the freeze-dried samples. In the control samples (Ctrl), the Cu, Ni, and Zn content was lower than 0.1% for each element. The presence of other elements, like C, O, P, S, Cl, Na, Mg, and Ca, in the samples was revealed by EDX analysis in Figure 1 and Figure 2. The highest metal content (*w*/*w*), considering the sum of the three metals, was found after exposure to the multimetallic solution, reaching a total value of 12.59%, with a Cu content 37.8% higher than the other metals. Nevertheless, considering the single metals, the uptake was lower compared to the monometallic solutions, suggesting a possible competition between metal ions for the same binding sites when multiple metals are present [29,30]. Indeed, the metal content in the samples obtained after exposure to Cu, Ni, and Zn monometallic solutions showed Cu, Ni, and Zn contents of 9.28, 7.91, and 5.42%, respectively, highlighting a higher affinity towards Cu. The specific interaction between Cu and the functional groups of RPS likely plays a key role in this high removal rate, as has already been demonstrated by different authors [6,31,32]. The lower metal uptake observed with Zn may suggest that the lower electronegativity of the metal (1.65 vs. 1.91/1.97 of Cu and Ni) affects metal uptake. Previous studies highlighted that the efficiency of metal biosorption is significantly influenced by the electronegativity of metal ions [33,34]. The retention and binding affinity of metals to biosorbents often involve electron-sharing interactions, which are stronger with metals of higher electronegativity. For example, biosorption studies with algae and other natural biosorbents found that metals with a higher electronegativity, like Cu and Zn, tend to form stronger bonds with biosorbent functional groups compared to less electronegative metals. This is due to a preference of these metals to engage in covalent bonding, which is more readily achieved with higher electronegativity, enhancing biosorption efficiency [35,36].

The observations of freeze-dried samples with SEM-EDX revealed the presence of a rougher surface in the multimetallic sample compared to the control, with clusters of particles attached to the RPS (Figure 1) indicating the successful adsorption of metals. The irregularities on the surface suggest strong interactions between the RPS and the metal ions, with visible accumulation zones (indicated by white arrows). The EDX spectra revealed the presence of Cu, Ni, and Zn, confirming the successful adsorption of all three metals (Figure 1). Cu had the highest atomic and weight percentages, followed by Ni and Zn. According to the ICP-OES results, these data confirm that RPS preferentially binds to Cu over the other two metals in a competitive environment.

To deeply investigate the effect of the metals on the morphological and surface features, the samples obtained after contact with monometallic solutions were observed by SEM-EDX (Figure 2). Interestingly, all the samples exhibited different morphological features compared to the control. Namely, the Cu-treated sample was characterized by a rough surface with densely packed metal deposits along the RPS strands (whiter zones indicated by the arrows). Similarly, the Ni-treated sample showed white particles on the surfaces of the RPS strands but were less uniformly distributed, while the surface morphology for the Zn-treated sample showed the least amount of change relative to the control. Fewer agglomerates were found bound to the RPS strand. Additionally, the EDX analysis exhibited a reduction in K and Mg content in the samples kept in contact with the metallic solutions (Figure 1 and Figure 2). Many authors have associated the variation in light-metal content (such as K, Mg, Ca) with an ion exchange mechanism [5,6]. Indeed, some cations can remain tightly bound to the biomass even after the pre-treatment before being exchanged by the heavy metals [6]. Ion exchange is considered one of the most common biosorption mechanisms and is influenced by the electronegativity and ionic radius of the metals [5,6]. The increase in Cl content (Figure 1 and Figure 2) can be explained by the supply of metals in their chloride form affecting the total Cl content of the solutions.

**Figure 3:**
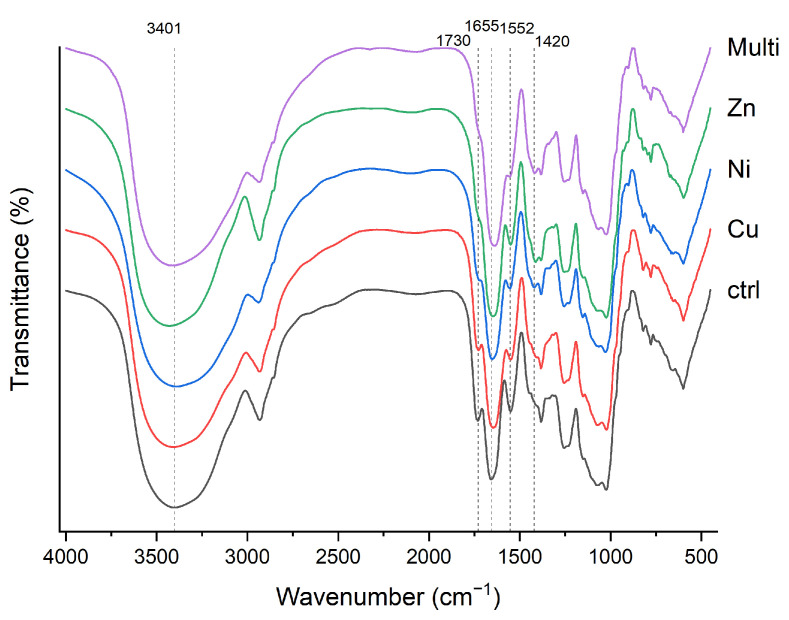
FT-IR spectra of RPS-containing supernatant after exposure to distilled water (ctrl) or monometallic and multimetallic solutions. Bands with major differences after metal exposure are highlighted with dotted lines.

The FT-IR spectra acquired on the samples were compared to hypothesize the functional groups of the RPS-containing supernatant and, particularly, those involved in the binding (Figure 3).

Table 2 summarizes the peak of the main bands in the spectra collected from the samples after exposure to metallic solutions or water (control). All spectra contain typical features of EPS, such as the broad band around 3400 cm^−1^, which is attributed to O-H stretching vibration [37,38,39], and the band around 2900 cm^−1^, which is assigned to the asymmetric stretching vibrations of C-H and C-H_2_ [39,40]. The strong band visible around 1600 cm^−1^ can be attributed to the stretching vibration of C=O and C-N (Amide I) that, together with the N-H/C-N stretch of Amide II at 1500 cm^−1^ [41], can be attributed to the presence of peptides and/or aminosugars in the supernatant. Also, the multiple bands detected between 1383 and 700 cm^−1^ are usually associated with the presence of polysaccharidic bones and sulfate groups [40,42,43]. The presence of sulfate groups is a typical feature of cyanobacterial polysaccharides. Indeed, they can constitute up to 20% of EPS dry weight, and they have been found in about 75% of analyzed EPS [3,4]. The comparison of the spectra (Figure 3, Table 2) revealed some modifications in the samples exposed to metallic solutions compared to the control. The differences include changes in band intensity, the appearance of new bands, and shifts in absorption bands, reflecting interactions with metallic ions [6,35,37]. Major differences were observed in O-H stretching, particularly in the Ni and Zn samples, where it moved from approximately 3401 to 3871 and 3429 cm^−1^, respectively. A new band, characteristic of C-H deformation in carboxyl groups [6], appeared around 1420 cm^−1^ in the samples exposed to Ni, Zn, and multimetallic solutions. The band at 1730 cm^−1^, assigned to C=O, disappeared after exposure to all metals except Cu. Small shifts were also detected in the Amide I and II bands (around 1655 and 1552 cm^−1^, respectively). The observed changes in FT-IR spectra suggest alterations in polymer conformation, with RPS functional groups, particularly hydroxyl and carbonyl groups, playing key roles in metal binding. Mota et al. [6], who analyzed the RPS of *Cyanothece* sp. CCY 0110 after exposure to various metallic ions, reported that sulfhydryl functional groups did not actively participate in metal absorption due to the absence of significant spectral shifts in their positions. Conversely, shifts in bands corresponding to C-H deformation and O-H stretching were noted. Similarly, Pagnucco et al. [37] observed changes in band intensity, shifts, and the appearance of new bands after metal exposure in different bacterial strains, indicating strain-specific alterations in cell surface properties induced by metal uptake. These findings highlight the role of strain-specific features in determining biosorption mechanisms.

**Figure 4:**
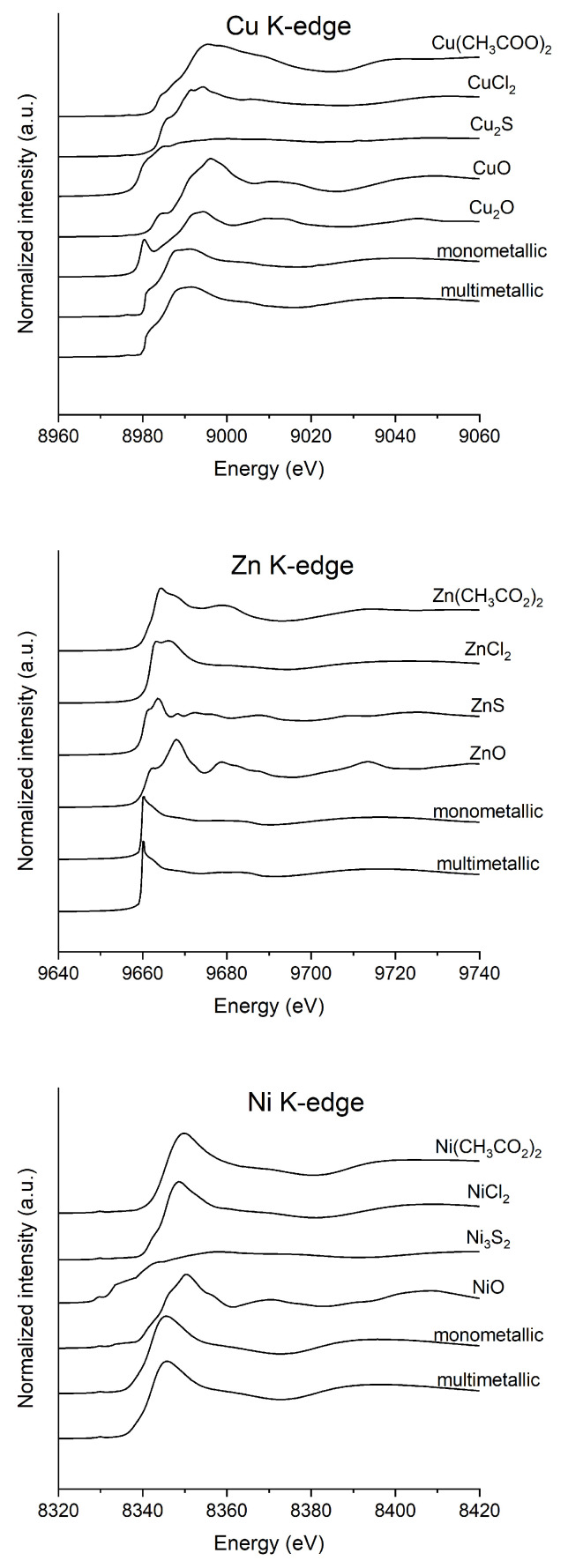
From the top: XANES spectra of measured samples and reference compounds at Cu K-edge, Zn K-edge, and Ni K-edge. For each sample, the average is 2–4 scans.

XAS data provided further insights into the coordination environment of each metal. The X-ray absorption near edge structure (XANES) spectra are presented for comparison in Figure 4. The comparison of the XANES spectra of metal-enriched samples with those obtained from the model compounds or found in the literature, suggests that the oxidation states of Cu, Zn, and Ni remained unchanged upon binding to the biosorbents, confirming their bivalent nature. These findings are consistent with those described in other studies on metal interactions with microbial biomasses and EPS using the same metals [45,46,47].

An EXAFS analysis was performed to identify the coordination environment of metals in the RPS-containing supernatant. EXAFS spectra in biological matrices are often complex due to (i) distorted geometries and multiple neighboring atoms (O, N, S, Cl) in the nearest shell, (ii) low atomic number elements (C, O, N) in the next-nearest coordination shells producing weak, overlapping signals, (iii) numerous multiple scattering paths within metal–organic complexes [48], and (iv) the impossibility of distinguishing elements with a similar Z, such as (C, O) or (S, Cl, P), since the scattering power is directly related to the atomic number of the elements. Also, coordination numbers derived from EXAFS have limited accuracy due to parameter correlations, particularly with the many-body reduction factor (S_0_^2^) and Debye–Waller factor (σ^2^), with an estimated accuracy of 20% [22].

**Figure 5:**
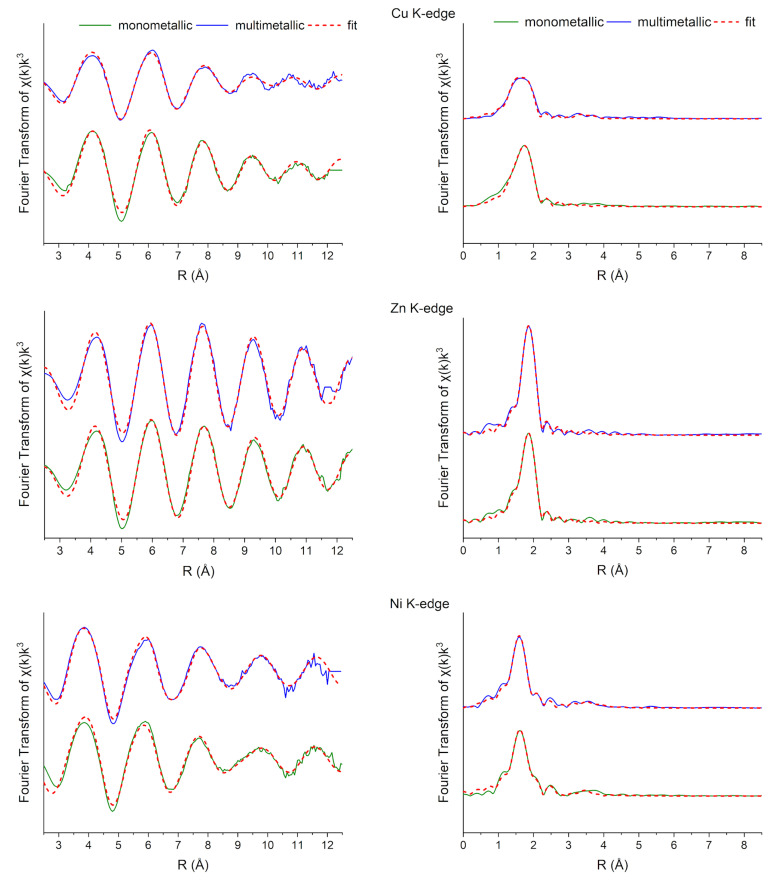
EXAFS (**on the left**) and Fourier-transformed (**on the right**) spectra of studied samples (continuous lines) and their fit (dotted red lines) at Cu, Zn, and Ni K-edge (from the top to the bottom). For each sample, the average is 2–4 scans.

The metal coordination environment in the RPS-containing supernatant of *Cyanothece* sp. CE4 reflected a mixed surrounding, mainly composed of (O, N) and (Cl, S) atoms in the first coordination shell. The initial fittings used one type of ligand in the first coordination shell with a fixed amplitude factor derived from model compounds letting the coordination number be free to vary. Subsequently, mixed coordination shells were modeled by adopting the bond valence method to analyze the atomic coordination ratio by valence [21], applying the procedure reported in [22]. This approach considers three potential scenarios: (i) binding of metals to both lighter (O, N) and heavier (Cl, S) elements within the same shell, (ii) binding to light and heavy elements in distinct coordination polyhedra, or (iii) a mix of these. The EXAFS and Fourier-transformed spectra of the measured samples are shown in Figure 5 together with the obtained multiparameter fits. The main EXAFS parameters obtained with all combinations tested are shown in Table 3.

The optimal fitting conditions for each sample were determined by analyzing the residuals in the EXAFS and Fourier-transformed spectra, as well as by examining the σ^2^ values and fitting metrics (reduced chi-squared and R-factor). Five distinct (O, N) and (Cl, S) configurations emerged: exclusive heavier elements (Cl, S) or lighter elements (O, N) in the first shell (ratios of zero or one, respectively); predominance of heavier elements within the same or different polyhedra (ratio ~ 0.25); equal proportions of heavier and lighter elements (ratio ~ 0.5); and predominance of lighter elements (ratio ~ 0.75). Despite testing all path combinations (whose fit details are illustrated in Table 3), the stronger Cl peak observed by EDX microanalysis after exposure to metallic solutions (Figure 1 and Figure 2) and the lower R-factor for fits carried out adopting (Cl, O) suggest that the O and Cl coordination paths are most probable, and therefore, only those fits will be discussed below for simplicity. Nevertheless, the presence of S and/or N cannot be excluded.

The nearest neighbors of Cu in mono- and multimetallic environments were 3.73–3.93 O atoms at 1.91–1.93 Å distance and 3.63–3.75 Cl atoms at 2.22–2.23 Å distance. The increase in the O and Cl coordination number after exposure to a multimetallic solution is related to the increase in the O/Cl ratio (from 0.16 to 0.34) and the necessity to satisfy the bond valence.

The nearest neighbors of Zn in a monometallic environment were 5.29 O atoms at 2.06 Å distance and 4.08 Cl atoms at 2.27 Å distance. The O/Cl ratio in this case was lower than 0.5, suggesting a greater involvement of Cl atoms in the coordination sphere, confirmed also by EDX analysis. In agreement, the O/Cl ratio drops to zero in a multimetallic environment, where only Cl contributed to EXAFS fitting in the first coordination shell at the same distance as the monometallic environment. The fitting carried out for the Ni spectra improved by adding the Ni-Cl path for the first coordination shell. The Ni results coordinated with 5.87–6.01 O atoms at 2.06–2.07 Å and 5.54–5.77 Cl atoms at 2.40–2.41 Å in an octahedron geometry. The O/Cl ratio slightly increased in a multimetallic environment from 0.93 to 0.96. The greater binding preference of Zn to Cl atoms compared to Cu and especially Ni can be attributed to the different electronic configurations of these metals, which are characterized by varying atomic numbers. Zn has a *d*^10^ configuration, resulting in no ligand field stabilization energy and associated stereochemical preferences [49]. Thus, the coordination number and geometry of zinc complexes are determined mainly by the radii of both the metal ion and the coordinating ligand [50]. This allows Zn to interact more readily with larger, softer ligands like Cl. In contrast, a Ni electron configuration leads to a higher ligand field stabilization energy, making it more inclined to bond with smaller, harder ligands such as oxygen, which aligns with its preference for stronger electrostatic interactions. This may also explain Ni’s tendency to adopt an octahedral geometry. Cu, on the other hand, occupies an intermediate position in this regard. The presence of Cl in all the samples, even those not in contact with metal solutions (Figure 1 and Figure 2), may be attributed to the acidic treatment performed prior to metal exposure, as well as the hypersaline conditions employed for cultivating *Cyanothece* sp. CE4. Indeed, this strain belongs to *Cyanothece* cluster 3, known as “*Halothece*”, within the order Chroococcales, which encompasses extreme halotolerant species [12,51]. Recent studies have highlighted the potential of halophilic cyanobacteria and microalgae for water biodesalination due to their ability to bind Cl− ions, facilitated by the sticky properties of extracellular polymeric substances (EPS) and their unique chemical composition [52]. Hence, it can be concluded that the halophilic behavior of *Cyanothece* sp. CE4 may significantly influence its metal biosorption potential and selectivity.

Fitting next-nearest coordination shells for the Cu, Ni, and Zn samples was challenging due to the significant disorder and weak, overlapping EXAFS signals from low atomic number elements (C, O, N). Previous studies have attempted to attribute similar EXAFS features to C, P, or S atoms at distances of 2.0–3.2 Å [46,47]. In this study, attempts at the fitting of residuals suggested the presence of Cu-Cu paths (*N* 4, 3.85–3.93 Å) and Cu-O (*N* 2–6, 2.30–2.38 Å) paths in the Cu samples and of Ni-Ni (*N* 2–4, 2.93–2.94 Å) and Ni-S (*N* 6, 4.24 Å) paths in the Ni samples.

### 3.2. Extracted RPS Experiment

**Figure 6:**
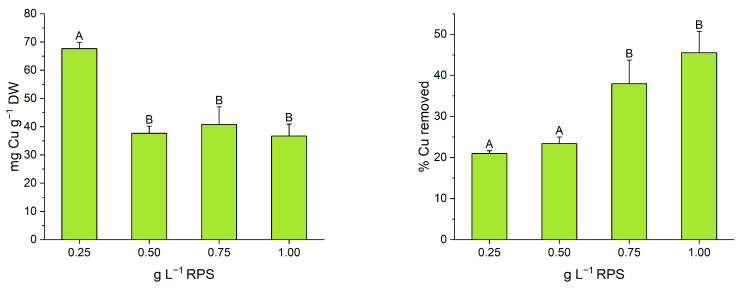
Specific Cu uptake (**on the left**) and Cu removal efficiency (**on the right**) by extracted RPS at different concentrations. Data are shown as mean ± SD (*n* = 3). Different letters mean statistically significant differences as determined by ANOVA test.

To confirm the role in metal biosorption of the RPS contained in the supernatant, in addition to the previous experiments performed with the whole supernatant, an experiment was carried out by adopting only the RPS extracted and characterized by a sulfate content of 26% (*w*/*w*) at varying concentrations to remove Cu. The results (Figure 6) showed a significantly higher specific Cu uptake (*p* < 0.0001) at the lowest tested RPS concentration (0.25 g L^−1^), achieving 67 mg Cu g^−1^ RPS DW. In contrast, the highest Cu removal efficiencies (*p* < 0.01) were observed at the highest RPS concentrations (0.75 and 1.00 g L^−1^), with a removal rate of up to 45%.

Notably, the maximum specific Cu uptake, corresponding to 1.05 mmol Cu g^−1^ DW, far exceeded the results reported by Mota et al. [6] who used the RPS from *Cyanothece* sp. CCY 0110 as a Cu biosorbent following an acidic pre-treatment. It also outperformed the findings by Concórdio-Reis et al. [9], who achieved a Cu removal efficiency and specific uptake of around 35% and 25 mg Cu g^−1^ DW, respectively, using 5 g L^−1^ of EPS secreted by the bacterium *Enterobacter* A47. Znou et al. [32] reported a significant increase in specific Cu uptake, increasing the concentration of the EPS from a mesophilic bacterium from 0.05 to 0.20 g L^−1^, reaching an uptake value of 62 mg Cu g^−1^ EPS DW. According to various studies, high concentrations of polysaccharides can increase the viscosity of biosorbents and enhance polymer–polymer interactions, which in turn, can hinder metal removal, as also observed in this study [9,53].

## 4. Conclusions

This study demonstrates that the supernatant collected from *Cyanothece* sp. CE4 cultures, containing released polysaccharides (RPS), can serve as an efficient biosorbent for heavy metals such as Cu, Ni, and Zn. The RPS-containing supernatant exhibited selective metal-binding capabilities, showing the highest affinity for Cu. Additionally, the presence of Cl, in addition to O, in the coordination shells—mainly for Zn—further underscores the strain-specific metal interaction capacities of *Cyanothece* sp. CE4, which can be attributable to its halophilic nature. The ability to use the supernatant alone for metal removal allows the remaining cyanobacterial biomass to be repurposed for other applications within a biorefinery. This dual utility highlights the potential of cyanobacterial cultures in circular economy models, offering sustainable solutions for both environmental remediation and resource recovery.

## Figures and Tables

**Table 1:** Cu, Ni, and Zn content (% *w*/*w*) in RPS-containing supernatant after contact with water (ctrl) or metallic solutions.

	Cu (%)		Ni (%)		Zn (%)	
	Average	St. Dev.	Average	St. Dev.	Average	St. Dev.
**Ctrl**	0.05	0.07	0.00	0.01	0.09	0.12
**Multi**	5.14	0.75	3.73	0.08	3.72	0.03
**Ni**	0.04	0.00	7.91	1.60	0.10	0.02
**Cu**	9.28	0.52	0.06	0.01	0.11	0.04
**Zn**	0.04	0.00	0.00	0.00	5.42	0.23

**Table 2:** FT-IR absorption bands and possible assignment for the control and metal-loaded samples.

Wave Number (cm^−1^)						
Ctrl	Cu	Ni	Zn	Multi	Assignment	References
3401	3406	3871	3429	3401	O-H	[37,38]
2929	2933	2938	2933	2938	C-H	[39,40,41]
1730	1726	nd	nd	nd	C=O	[41]
1655	1646	1651	1646	1642	C=O. C-N	[34,38,41]
1552	1548	1552	1552	1557	N-H. C-N	[6,38,44]
nd	nd	1422	1412	1417	C-H	[6,42]
1384	1384	1384	1382	1382	C-H. S=O	[6,41,42,43]
1253	1256	1255	1255	1256	C-O	[41,42,43]
1155	1152	1153	1153	1153	C-O-C	[39]
1075	1075	1075	1075	1071	C-O	[42]
1025	1025	1026	1025	1026	C-O-S. C-O-C. C-O. C-C	[41,42,43,44]
821	820	819	818	816	C-O-S. C-O-C. C-O. C-C	[41,42,43,44]
780	779	778	778	778	C-O-S	[41,42,43]
599	599	599	599	599	C-H	[38]

nd means not detected; wave numbers (cm^−1^) were determined at the central point of the band.

**Table 3:** EXAFS multiparameter fit details for the studied samples.

Solution	*k* Range (Å^−1^)	Path	*R* (Å)	*N*	*v_i_*	(O, N):(Cl, S)	*σ^2^* (Å^−2^)	R-Factor
Cu	3.0–12.6	Cu-O	1.91 (5)	3.73	0.54	0.16 (24)	0.001 (8)	0.013
		Cu-Cl	2.22 (2)	3.63	0.55		0.003 (4)	
		Cu-O	1.98 (5)	4.52	0.44	0.69 (23)	0.009 (0)	0.018
		Cu-S	2.26 (2)	5.94	0.34		0.004 (3)	
		Cu-N	2.01 (5)	5.82	0.34	0.71 (25)	0.009 (0)	0.026
		Cu-S	2.27 (3)	6.02	0.33		0.004 (4)	
		Cu-N	1.91 (2)	4.52	0.44	0.10 (29)	0.008 (3)	0.023
		Cu-Cl	2.22 (4)	3.39	0.58		0.008 (3)	
Cu (multi)	2.9–12.3	Cu-O	1.93 (3)	3.93	0.51	0.34 (24)	0.003 (4)	0.008
		Cu-Cl	2.23 (2)	3.75	0.53		0.008 (2)	
		Cu-O	1.96 (2)	4.34	0.46	0.71 (40)	0.008 (3)	0.016
		Cu-S	2.26 (4)	5.96	0.34		0.006 (7)	
		Cu-N	1.91 (4)	4.60	0.43	0.19 (29)	0.001 (7)	0.017
		Cu-S	2.22 (2)	5.35	0.37		0.011 (2)	
		Cu-N	1.94 (2)	4.85	0.41	0.26 (17)	0.001 (3)	0.009
		Cu-Cl	2.22 (2)	2.67	0.54		0.008 (1)	
Zn	3.2–12.8	Zn-O	2.06 (7)	5.29	0.38	0.39 (23)	0.010 (2)	0.008
		Zn-Cl	2.27 (2)	4.08	0.49		0.004 (1)	
		Zn-O	2.09 (4)	5.73	0.35	0.47 (19)	0.006 (2)	0.013
		Zn-S	2.31 (1)	3.58	0.56		0.003 (1)	
		Zn-N	2.08 (4)	4.63	0.43	0.36 (26)	0.002 (3)	0.015
		Zn-S	2.29 (2)	3.45	0.58		0.002 (1)	
		Zn-N	1.98 (12)	3.57	0.56	0.12 (32)	0.001 (11)	0.010
		Zn-Cl	2.26 (1)	3.88	0.52		0.005 (2)	
Zn (multi)	3.2–12.8	Zn-Cl	2.27 (0)	3.97	0.49	0.00	0.005 (0)	0.002
		Zn-S	2.28 (0)	4.40	0.60	0.00	0.005 (0)	0.003
Ni	2.8–12.0	Ni-O	2.07 (2)	6.01	0.33	0.93 (10)	0.007 (2)	0.007
		Ni-Cl	2.40 (3)	5.54	0.36		0.001 (8)	
		Ni-O	2.07 (2)	5.98	0.33	0.94 (9)	0.007 (1)	0.007
		Ni-S	2.40 (3)	7.10	0.28		0.001 (9)	
		Ni-N	2.06 (2)	6.30	0.32	0.57 (19)	0.003 (2)	0.008
		Ni-Cl	2.32 (2)	4.47	0.45		0.007 (3)	
		Ni-N	2.06 (1)	6.28	0.32	0.55 (20)	0.003 (1)	0.008
		Ni-S	2.33 (2)	5.76	0.35		0.009 (4)	
Ni (multi)	2.8–12.0	Ni-O	2.06 (2)	5.87	0.34	0.96 (9)	0.007 (1)	0.005
		Ni-Cl	2.41 (3)	5.77	0.35		0.000 (9)	
		Ni-O	2.07 (2)	5.97	0.34	0.99 (8)	0.008 (1)	0.006
		Ni-S	2.43 (3)	7.54	0.27		0.018 (11)	
		Ni-N	2.06 (1)	6.14	0.33	0.69 (14)	0.003 (2)	0.004
		Ni-Cl	2.33 (2)	4.57	0.44		0.007 (3)	
		Ni-N	2.06 (1)	6.13	0.33	0.68 (17)	0.003 (5)	0.004
		Ni-S	2.34 (4)	5.91	0.34		0.008 (3)	

Notes: *S*_0_^2^ (many-body amplitude reduction factor) was fixed to 0.80 and 0.90 for Cu and Zn samples, respectively, on the basis of the fitting carried out on reference compounds, and to 0.99 for Ni-samples, on the basis of the fitting carried out on other cyanobacterial samples with only oxygen as ligand. *N* = path degeneracy. *v_i_* = valence. *R* = path length. *σ*^2^ = Debye–Waller factor. Uncertainties of the fit shown within parenthesis.

## Data Availability

The raw data supporting the conclusions of this article will be made available by the authors upon request or are available online at the following link: https://doi.org/10.15151/ESRF-ES-1234126108 Ciani, M., Lepore, G. O., and Margheri, S. (2026). Chemical and structural characterization of metal-organic materials obtained through heavy metal biosorption by exopolysaccharide-producing [Dataset]. European Synchrotron Radiation Facility.
